# Coherent anti-Stokes Raman scattering cell imaging and segmentation with unsupervised data analysis

**DOI:** 10.3389/fcell.2022.933897

**Published:** 2022-08-16

**Authors:** Damien Boildieu, Tiffany Guerenne-Del Ben, Ludovic Duponchel, Vincent Sol, Jean-Michel Petit, Éric Champion, Hideaki Kano, David Helbert, Amandine Magnaudeix, Philippe Leproux, Philippe Carré

**Affiliations:** ^1^ University of Limoges, CNRS, XLIM, UMR 7252, Limoges, France; ^2^ University of Poitiers, CNRS, XLIM, UMR 7252, Poitiers, France; ^3^ University of Limoges, PEIRENE, UR 22722, Limoges, France; ^4^ University of Lille, CNRS, UMR 8516, LASIRE - Laboratoire de Spectroscopie Pour Les Interactions, La Réactivité et L’Environnement, Lille, France; ^5^ University of Limoges, CNRS, Institut de Recherche sur Les Céramiques, UMR 7315, Limoges, France; ^6^ Department of Chemistry, Faculty of Science, Kyushu University, Fukuoka, Japan

**Keywords:** cell imaging, cell segmentation, coherent anti-Stokes Raman scattering, unsupervised data analysis, coherent Raman imaging, label-free imaging, supercontinuum, multivariate curve resolution

## Abstract

Coherent Raman imaging has been extensively applied to live-cell imaging in the last 2 decades, allowing to probe the intracellular lipid, protein, nucleic acid, and water content with a high-acquisition rate and sensitivity. In this context, multiplex coherent anti-Stokes Raman scattering (MCARS) microspectroscopy using sub-nanosecond laser pulses is now recognized as a mature and straightforward technology for label-free bioimaging, offering the high spectral resolution of conventional Raman spectroscopy with reduced acquisition time. Here, we introduce the combination of the MCARS imaging technique with unsupervised data analysis based on multivariate curve resolution (MCR). The MCR process is implemented under the classical signal non-negativity constraint and, even more originally, under a new spatial constraint based on cell segmentation. We thus introduce a new methodology for hyperspectral cell imaging and segmentation, based on a simple, unsupervised workflow without any spectrum-to-spectrum phase retrieval computation. We first assess the robustness of our approach by considering cells of different types, namely, from the human HEK293 and murine C2C12 lines. To evaluate its applicability over a broader range, we then study HEK293 cells in different physiological states and experimental situations. Specifically, we compare an interphasic cell with a mitotic (prophase) one. We also present a comparison between a fixed cell and a living cell, in order to visualize the potential changes induced by the fixation protocol in cellular architecture. Next, with the aim of assessing more precisely the sensitivity of our approach, we study HEK293 living cells overexpressing tropomyosin-related kinase B (TrkB), a cancer-related membrane receptor, depending on the presence of its ligand, brain-derived neurotrophic factor (BDNF). Finally, the segmentation capability of the approach is evaluated in the case of a single cell and also by considering cell clusters of various sizes.

## 1 Introduction

In the context of label-free cell imaging, vibrational spectroscopic approaches have proven their effectiveness to visualize the cellular content and processes (Matthäus et al., 2008; Klein et al., 2012; Palonpon et al., 2013; Smith et al., 2016). As chemically selective techniques, they can provide both structural and functional information from the sample. Among these techniques, coherent anti-Stokes Raman scattering (CARS) and stimulated Raman scattering (SRS) have been extensively applied to live-cell imaging in the last 2 decades (Zumbusch et al., 1999; Freudiger et al., 2008; Cheng and Xie, 2015). More specifically, both methods have been successfully used to probe the intracellular lipid, protein, nucleic acid, and water content with a high-acquisition rate and sensitivity.

In this context, multiplex CARS (MCARS) microspectroscopy (Kee and Cicerone, 2004; Kano and Hamaguchi, 2005) is a powerful technology for extracting rich vibrational information from biological samples. It is based on the excitation of the sample by two laser beams, namely, the monochromatic pump (ω_p_ frequency) and the broadband Stokes (ω_S_ frequency) beam. When the difference ω_p_ - ω_S_ matches the vibration frequency of a vibrational mode, a resonant CARS signal is generated at the frequency 2ω_p_ - ω_S_, that is, at a wavelength lower than that of the pump laser, preventing any fluorescence background in the measured MCARS spectrum. This is a considerable advantage for this technique because conventional Raman often presents a fluorescence that completely masks the vibrational signal, especially in biology. However, it is well-known that MCARS is affected by the presence of a so-called non-resonant background (NRB), which is inherent to its physical mechanism. In this regard, phase retrieval approaches like the maximum entropy method (MEM) and time-domain Kramers–Kronig (TDKK) are generally used to extract the pure vibrational signal and to recover conventional Raman-like spectra (Cicerone et al., 2012). These spectra are then more easily interpretable and allow generating high-contrast images from the identified Raman bands. More recently, phase extraction from MCARS spectra was investigated by using a deep learning approach, based on the concept of supervised methods and using a long short-term memory network (LSTM) architecture (Houhou et al., 2020). Another study introduced a workflow for fast Raman signal extraction, denoising, error correction, and the applicability of this workflow to machine learning (Camp et al., 2020).

On the other hand, chemometric methods as principal component analysis (PCA) or multivariate curve resolution (MCR) are widely used in spectroscopy-based data analysis (Pisapia et al., 2018; Ghaffari et al., 2019; Amigo, 2020). They consist of an unsupervised, statistical multivariate exploration of the collected data, being considered as established approaches, especially in the field of Raman microspectroscopy. PCA’s purpose is to project data into a subspace, allowing to decorrelate the measurements and to maximize the variance of projections. Thus, PCA facilitates the observation of the relevant information of the dataset. Following this concept of projection of a multicomponent signal into subspaces, MCR is an iterative matrix decomposition method. It decomposes the considered dataset by means of its projection into a subspace guided by different constraints (for instance, non-negativity constraint). This is a definite advantage because this signal unmixing approach allows us to find contributions in the sample that are far more easily interpreted than those obtained from PCA. Specifically, the MCR approach decomposes the initial dataset into a matrix of “pure concentration profiles” and a matrix of “pure spectral profiles,” providing a concentration map (quantitative information) and the associated spectrum (qualitative information) of each contribution within the investigated sample. For example, the algorithm allows, in a context of chemical analysis, to extract the “pure” chemical species of the sample (De Juan and Tauler, 2021).

To date, MCR has never been applied to the analysis of MCARS hyperspectral datasets. The only work that could come close to such an approach was published by Zhang et al., 2013, with idea to extract images of major components in breast cancer cells using SRS. In this case, the multivariate exploration involved a spectral stack of only ∼80 SRS images, and representative spectra of lipid droplets, nuclei, and culture medium were used at the initial estimation step (supervised method). Regarding CARS data processing in the context of cell imaging, few studies report on the use of chemometric methods within the proposed workflow, including PCA (Parekh et al., 2010; Pohling et al., 2011; Lee et al., 2014), singular value decomposition (SVD) (Masia et al., 2013), or hierarchical cluster analysis (HCA) (El Mashtoly et al., 2014). These studies aim at performing an unsupervised quantitative chemical analysis of an unknown biological sample and/or a classification of cells or subcellular organelles. Overall, they rely on relatively complex data analysis pipelines, including a heavy spectrum-to-spectrum phase retrieval step, which limit their dissemination into the biomedical field. A more recent work focused on using PCA and k-means clustering to perform a comparison between CARS and SRS in the context of tissue imaging (Bocklitz et al., 2018). Based on all these works, it is evident that an MCR-based approach should be able to bring a new look on MCARS data by generating less biased cell images while facilitating the interpretation of the extracted chemical and biological information. Moreover, such approach would be all the more powerful as it could take both the spectral and spatial dimensions of the dataset into account, and the sole spectra being usually exploited in the field.

Today, MCARS microspectroscopy using a dual-output sub-nanosecond laser source is recognized as a mature and straightforward technology for bioimaging, offering the high spectral resolution of conventional Raman spectroscopy with reduced acquisition time (Kano et al., 2019; Kaneta et al., 2021). We have recently demonstrated its efficiency for cell cycle studies (Guerenne-Del Ben et al., 2019) or cancer cells characterization (Guerenne-Del Ben et al., 2020). In the proposed study, we naturally combine the potential of both this imaging technique and the MCR source separation method under the classical signal non-negativity constraint and, even more originally, under a new spatial constraint based on cell segmentation. We thus introduce a new approach of MCARS hyperspectral cell imaging and segmentation, based on a simple workflow, without any phase retrieval computation, for data acquisition, chemical analysis, and visualization.

The imaging capability of this approach is first described using a MCARS dataset obtained from a fixed human embryonic kidney 293 (HEK293) cell in the interphase of the cell cycle (G_1_/S). This cell is subsequently used as the “reference” cell. Second, we compare the results obtained for the reference HEK293 cell with those obtained for a premyoblastic cell from the C2C12 murine line under similar conditions (*i.e.*, fixed during the interphase of the cell cycle). This comparison allows us to assess the robustness of our approach by considering two very different cell types. To evaluate its applicability over a broader range, we then study HEK293 cells in different physiological states and experimental situations (Guerenne-Del Ben et al., 2019; Guerenne-Del Ben et al., 2020). For this purpose, we compare the results of the reference (interphasic) cell to those obtained from another cell in mitosis (prophase), during which important nuclear and cytoplasmic rearrangements occur. We also present a comparison between the reference (fixed) cell and a living cell, in order to visualize the potential changes induced by the fixation protocol in cellular architecture. Next, with the aim of assessing more specifically the sensitivity of our approach, we study HEK293 living cells overexpressing tropomyosin-related kinase B (TrkB), a cancer-related membrane receptor, depending on the presence of its ligand, brain-derived neurotrophic factor (BDNF). Finally, the application of the segmentation constraint in the MCR framework is introduced and discussed in the case of the single reference cell. Then, the segmentation capability of the approach is evaluated by considering cell clusters of various sizes.

## 2 Materials and methods

### 2.1 Cell samples preparation

The datasets of HEK293 cells used in this study were obtained from cell samples prepared by T. Guerenne-Del Ben, according to the protocols described in Guerenne-Del Ben et al., 2019 and Guerenne-Del Ben et al., 2020. HEK293 cell line was provided by Pr. F. Lalloue, CAPTuR, UMR INSERM 1308, Faculty of Medicine, University of Limoges, France. HEK293 cells were non-modified or overexpressing the TrkB receptor.

Non-modified HEK293 cells were routinely cultured in a complete medium consisting in high-glucose (4.5 g/L) DMEM (Gibco), supplemented with 10% (v/v) fetal calf serum (Eurobio), 1 μg/ml amphotericin B (Gibco), 100 units/ml penicillin, and 100 μg/ml streptomycin (Gibco) in an incubator heated at 37°C under 5% CO_2_ humidified atmosphere. HEK293 cells overexpressing TrkB were cultured in a complete medium supplemented with 750 μg/ml geneticin (G418, Roth). C2C12 cell line (ATCC, Manassas, VA, United States) was cultured in DMEM supplemented with l-glutamine, 10% (v/v) fetal calf serum, 50 units/mL penicillin, and 50 μg/ml streptomycin.

For MCARS analysis, the cells were seeded at a density of 8,750 cells/cm^2^ on 18-mm glass coverslips in 12-well plates for 48 h.

For the synchronization of non-modified HEK293 cells (study of the cell cycle), a first blocking with thymidine was performed for 18 h. Then for obtaining G_1_/S or mitotic cells, after a release phase in complete medium of, respectively, 9 and 4 h, the cells were exposed to either 2-mM thymidine (double block procedure) for 17 h or 100 ng/ml nocodazole (Sigma-Aldrich) during 12 h before being replaced in fresh complete medium (for more details, refer to Guerenne-Del Ben et al., 2019). When a fixation was performed, the cells were washed three times in DPBS (Gibco), immersed in 4% paraformaldehyde (PFA) for 10 min at room temperature, and washed again three times in DPBS. A subset of living cells was labeled with the nuclear dye Hoechst 33342 (ThermoFisher) at 10 μg/ml for 15 min and room temperature before being rinsed in DPBS.In addition, a subset of fixed cells was labeled with the nuclear dye DAPI (4′,6-diamidino-2-phenylindole, Sigma-Aldrich) at 1 μg/ml for 5 min.

HEK293 cells overexpressing TrkB were cultured in the presence of recombinant human BDNF (Peprotech) at a final concentration of 75 ng/ml, or its solvent (DPBS), for 72 h. Cell nuclei were then stained with Hoechst 33342 as described earlier.

Glass coverslips with living or fixed cells were mounted on a microscope slide in DPBS and sealed with nail polish.

### 2.2 MCARS microspectroscopy and fluorescence imaging

The MCARS microspectroscope and associated experimental conditions are described in Guerenne-Del Ben et al., 2019. In brief, the MCARS setup is based on a passively Q-switched microchip laser (Horus Laser, 1,064 nm, 1 ns, 20 kHz), a photonic crystal fiber for generating the broadband infrared Stokes wave, focusing (Olympus, UPlanSApo 60x, N.A. = 1.2, water immersion) and collecting (Nikon, S Plan Fluor ELWD 60x, N.A. = 0.7) microscope objectives, and a spectrometer (Horiba, LabRam HR Evolution). CARS spectra were acquired from 2,500 to 3,200 cm^−1^ with ∼0.8 cm^−1^ spectral resolution, 50 ms pixel dwell time, and 300 nm lateral step for cross-sectional mapping. The lateral and axial resolutions were ∼300 nm and ∼2 μm, respectively. The laser power of the pump and Stokes radiations at the sample position was 55 and 9 mW, respectively, for which no morphological change of cells was observed during the experiments.

Fluorescence imaging was realized on the same system by using a halogen light source, appropriate excitation and emission filters, and a dedicated CCD camera (Thorlabs, 1500M-GE).

### 2.3 Maximum entropy method

As we have seen in the introduction, CARS spectra suffer from the presence of NRB that greatly distorts the acquired spectra and therefore the images that are extracted afterward. For the purpose of spectroscopic validation of our approach, we used the MEM algorithm from Vartiainen et al. (2006) as a reference method to extract the imaginary part of the third order non-linear susceptibility (Im{*χ* ^(3)^}) and thus obtain conventional Raman-like spectra. The implementation of MEM was performed using MATLAB software (R2018b, MathWorks).

### 2.4 Multivariate curve resolution

**FIGURE 1:**
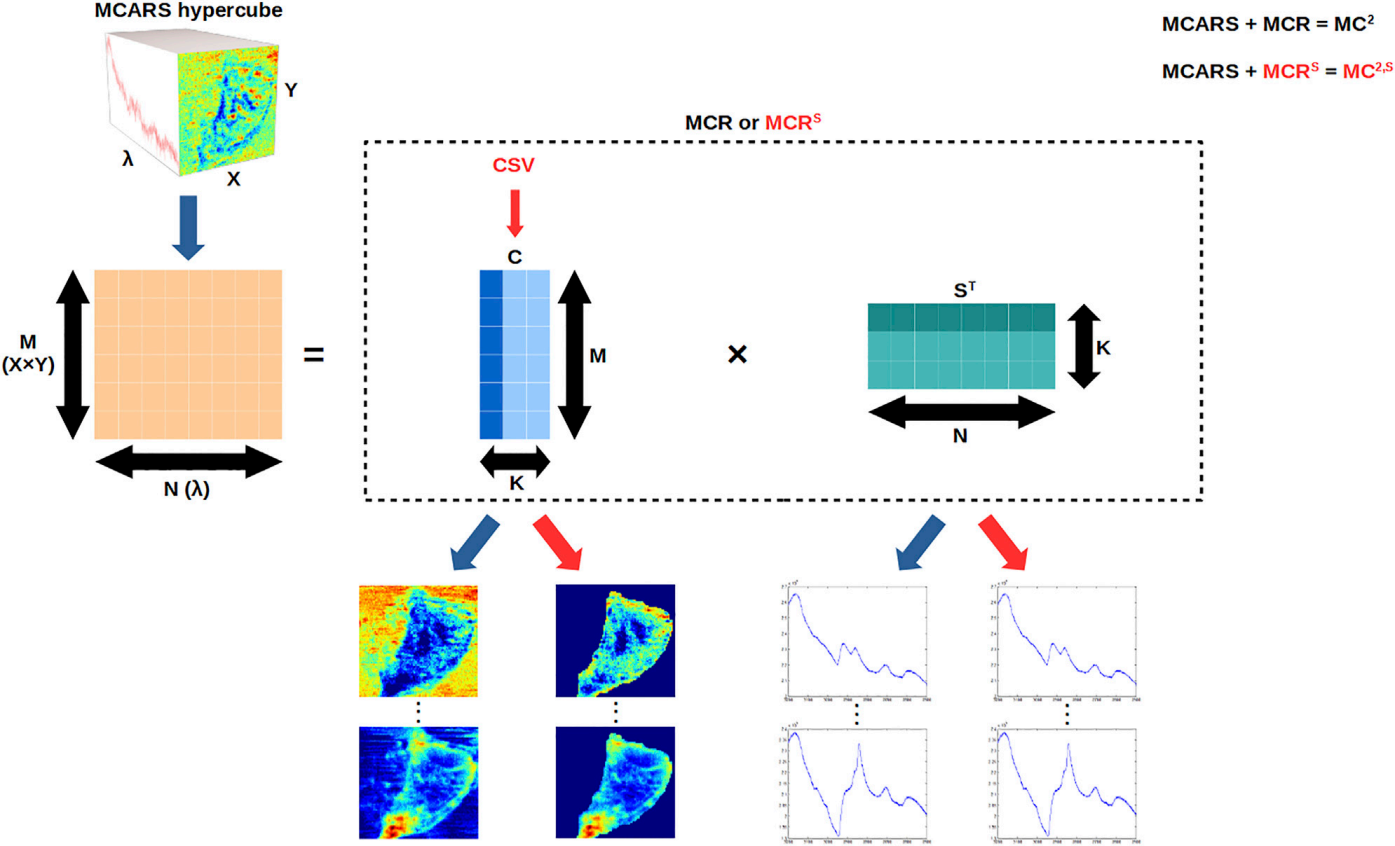
MC^2^ and MC^2,S^ frameworks. MCARS data are linearized, and then MCR or MCR^S^ is applied to compute the concentration M × K -matrix C and the spectra K × N -matrix $S^{T}$ iteratively. Finally, an image (reconstructed from C ; segmented in the case of MCR^S^) and a spectrum (stored in $S^{T}$ ) are available for each of the K extracted components. CSV stands for the Chan–Sandberg–Vese method, which is used as a segmentation constraint in MCR^S^.

MCARS datasets were analyzed using MCR (De Juan et al., 2014). MCR is a signal unmixing method, which aims at finding the K components of a multicomponent signal. The data matrix D , which contains M measures for N Raman shifts, is decomposed into two matrices, as depicted in Figure 1: the concentration M × K -matrix C , including the K component concentration maps along its columns (corresponding to the projection of the initial data), and the spectra K × N -matrix $S^{T}$ , including the K component spectra along its rows (corresponding to the projection basis). More formally, the model is defined as follows:

$$D=CS^{T}+E,$$ (1)

where E being the error matrix, containing data not represented by the basis and expected to be noise or irrelevant information. In the case of chemical mixture analysis, $c_{i,j}$ is the concentration of compound *j* for measuring (i.e. pixel) *i* and row *j* of $S^{T}$ , which is the characteristic spectrum of this compound.

In order to evaluate the quality of the MCR decomposition according to the value of K , the so-called lack-of-fit (LOF) was used:

$$LOF=\frac{\sum_{i,j}e_{i,j}^{2}}{\sum_{i,j}d_{i,j}^{2}},$$ (2)

where *e*_*i,j*_ and *d*_*i,j*_ being elements of row *i* and column *j* of matrices E and D , respectively. The LOF corresponds to the ratio between the data contained in E (i.e., not expressed by the model) and those contained in the experimental dataset. Based on the variation of the LOF as a function of K for the datasets used in our study, the number of components was set to K=5 . This choice will be investigated later in the Discussion.

Even if MCR allows us to explore complex signals, this approach nevertheless presents what is called ambiguities, meaning that different pairs of C and $S^{T}$ matrices can be acceptable solutions. In this context, applying constraints to C and $S^{T}$ during MCR calculation allows reducing these ambiguities and the number of possible solutions, in order to potentially converge toward a unique decomposition. Two of the most common constraints are non-negativity and normalization. The non-negativity constraint ensures to have only positive values for all elements of C and/or $S^{T}$ matrices, while the normalization constraint implies that the sum of all values of each row or column of C and/or $S^{T}$ is equal to one. Obviously, constraints have to be selected according to the acquisition method, the study subject, and the intrinsic nature of the data. In this study, we applied the non-negativity constraint to the concentration matrix C and to the spectra matrix $S^{T}$ , while the normalization constraint was only applied to each row (i.e., measure) of the concentration matrix.

One way to solve the MCR problem is to use an alternating regression algorithm to compute C and $S^{T}$ . Here, we used the common “alternating least squares” (ALS) algorithm, the method being then called MCR-ALS. Moreover, we applied the non-negativity constraint within the decomposition process by using non-negative least squares (NNLS) (Lawson and Hanson, 1974). Practically, these results are a three-step process: first, the C matrix is computed by NNLS, then it is normalized and, in turn, the S matrix is computed by NNLS. This process is then repeated until convergence. Furthermore, we used the SIMPLISMA algorithm (Windig and Guilment, 1991) for computing the initial S matrix of the iterative process.

We implemented the whole MCR-ALS workflow in Python language, based on the freely available “pyMCR” package (Camp, 2019). In the following sections, the combination of MCARS acquisition and MCR data analysis (with the two classical constraints) will be denoted as MC^2^ (Figure 1).

### 2.5 New cell segmentation constraint (MCR^S^)

To extract cells from their environment and refine the intracellular analysis, we have developed in this work a new spatial constraint to be applied to the concentration matrix C , additionally to the previous constraints in the MCR framework. Specifically, we applied a segmentation constraint based on the Chan–Sandberg–Vese (CSV) method (Chan et al., 2000), which has already shown its ability to segment biological cells (Dufour et al., 2005; Maška et al., 2013). With the use of this additional spatial constraint, the framework will be denoted as MCR^S^ and the whole approach as MC^2,S^ in this work (Figure 1).

The CSV method is an iterative segmentation method based on active contours. It consists of splitting the given image into two regions, using an energy-dependent model. The goal of the algorithm is to minimize the fitting energy between the model and the input image I :

$$arg\underset{c_{1},c_{2},C}{min}F\left(c_{1},c_{2},ℭ\right)$$ (3)

with $F\left(c_{1},c_{2},ℭ\right)=\mu \cdot Length\left(ℭ\right)+\nu \cdot Area\left(inside\left(ℭ\right)\right)+\lambda_{1}\int_{x,y\epsilon inside\left(ℭ\right)}||I\left(x,y\right)-c_{1}{||}^{2}dxdy+\lambda_{2}\int_{x,y\epsilon outside\left(ℭ\right)}||I\left(x,y\right)-c_{2}{||}^{2}dxdy,$

where ℭ is the border between the two regions, and *c_1_* and *c_2_* are the region averages. This equation is solved using a partial differential equation. The method requires the following parameters to be defined:

1) “Length penalty” *µ* (to be set between 0 and 1) regularizes the length of ℭ . It is the most important parameter to tune (Getreuer, 2012). A small value leads to define the border of the cells with potentially a high level of detail, while a large one generates a smoother boundary. Here, we use µ = 0.35. The rationale behind this choice is stated in Section 3 (more specifically, in the sub-section entitled “Comparison between MC^2^ and MC^2,S^ methods applied to the reference cell”;
2) “Area penalty” *ν* tunes the penalty (*ν* > 0) or reward (*ν* < 0) to be applied to the area inside ℭ during minimization. As we do not have any *a priori* on the size of cells in the acquisition window, *ν* is set to zero;
3) “Fit weights” *λ_1_* and *λ_2_* allow to control deviation from the original image in terms of pixel intensity for areas inside and outside ℭ , respectively. We use *λ_1_* = *λ_2_* = 1 in order to not to penalize one area against the other;
4) “Time step” Δt is the discretization step used to solve the partial differential equation. A small value gives better results with increased computation time, while a large value can lead to convergence problems. We use the default value Δt = 0.5 (Getreuer, 2012).

The new algorithm corresponding to one iteration of MCR^S^ is the following:

1) compute C using NNLS;
2) refold C into *K* images of size *X×Y* (consistently with the initial dataset);
3) apply the CSV method to these images in order to compute a segmentation mask;
4) use the mask to set concentration values outside ℭ to zero;
5) unfold segmented images in order to retrieve a (spatially) constrained C matrix;
6) apply the normalization constraint to C ;
7) compute S using NNLS.

The addition of the segmentation constraint to the MCR-ALS framework was also implemented in Python.

### 2.6 Projection onto reference spectra

In the case of living HEK293 cells overexpressing TrkB cancer-related protein, a particular approach was used to compare cells that were incubated with or without BDNF. First, the MC^2^ approach was applied to a cell that was incubated without BDNF, considering the spectra of the extracted components as reference spectra. Then, the MCARS dataset of a second cell, incubated with BDNF, was projected onto the basis of reference spectra by using the NNLS regression algorithm. The resulting concentration matrix was then normalized, as previously. With this approach, we could visualize the spatial distribution of the same components in both cells, comparatively.

### 2.7 Statistical analysis of cell segmentation

In order to generate the ground truth masks, bright-field images of the cells were opened within NIH ImageJ software (https://imagej.nih.gov/ij/) and manually segmented using the “Polygons selection” tool. The comparison between ground truth and MC^2,S^ masks, and the calculation of Dice similarity coefficients were realized by using “imshowpair” and “dice” functions in MATLAB software (R2018b, MathWorks).

The statistical analysis of Dice coefficients was performed using PAST software (O. Hammer, D.A.T. Harper, P.D. Ryan, PAST: paleontological statistics software package for education and data analysis, Palaeontol. Electron. 4 (2001) 1–9). After having tested the normality of the values using a Shapiro–Wilk test, a one-way analysis of variance (ANOVA) was performed and followed by a Tukey’s multiple comparison test. Differences were considered significant for *p* < 0.05.

## 3 Results

### 3.1 Spectroscopic validation of the approach

**FIGURE 2:**
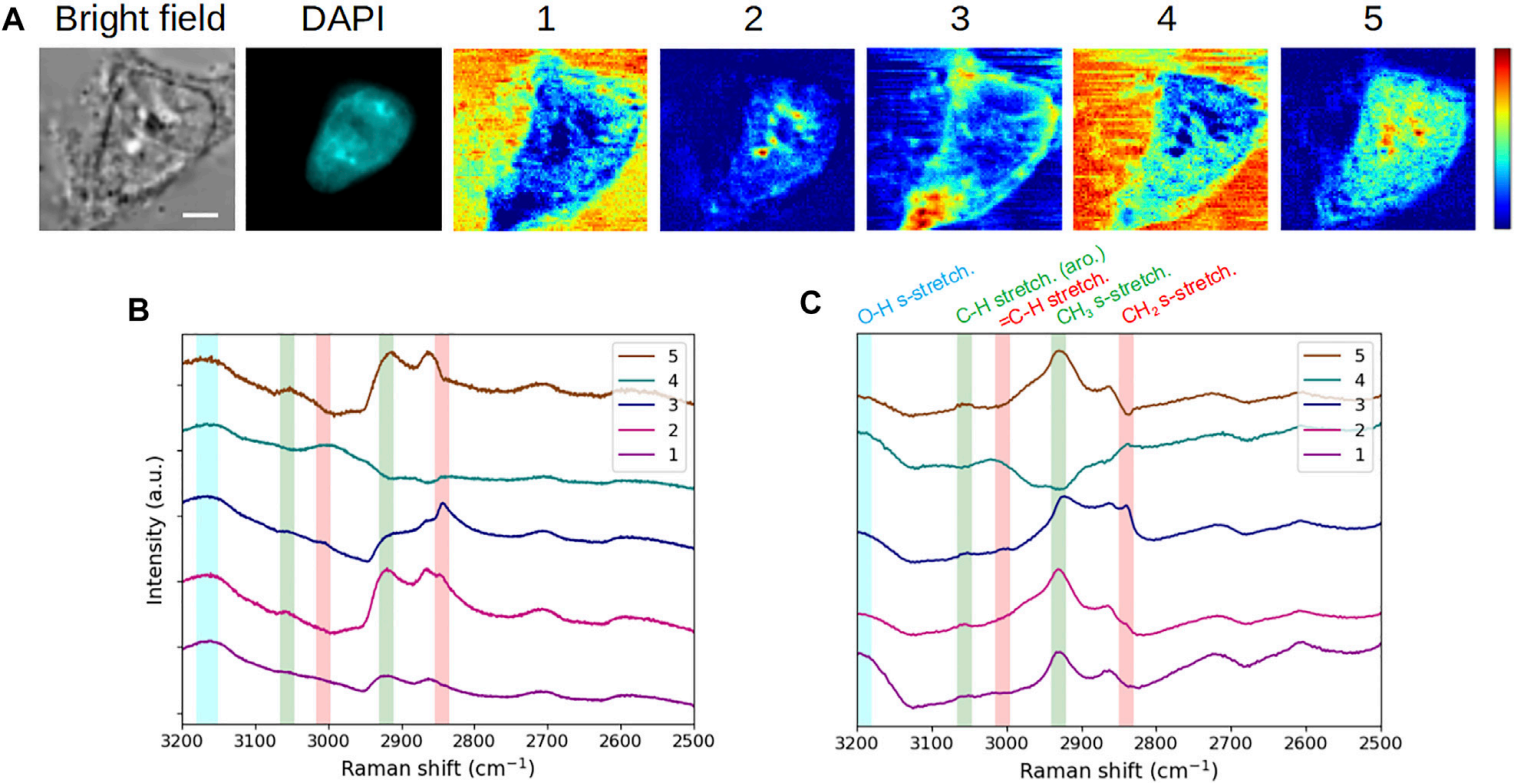
Application and spectroscopic validation of the MC^2^ approach for the analysis of a fixed interphase HEK293 cell. (**A)** Bright-field and fluorescence images and concentration maps obtained for the five computed components. The scale bar corresponds to 5 μm and applies to all images. **(B)** Spectra extracted for these five components. **(C)** Raman-like spectra after MEM processing, with the following vibrational modes highlighted: CH_2_ symmetric (2,845 cm^−1^) and = C-H (3,005 cm^−1^) stretching mainly associated to lipids (red vertical bars), CH_3_ symmetric (2,930 cm^−1^) and C-H aromatic (3,056 cm^−1^) stretching mainly associated to proteins (green vertical bars), and O-H symmetric stretching (3,200 cm^−1^) of water (blue vertical bar). Vertical bars were replicated into **(B)**, taking into account the peak spectral shift that occurs between raw CARS and MEM-processed data. After fixation, the cell was stained with DAPI.

We first applied the MC^2^ approach to the analysis of a fixed, DAPI-stained, interphase HEK293 cell. Figure 2A displays the bright-field and fluorescence images, as well as the five concentration maps, while the corresponding component spectra are plotted in Figure 2B. In order to validate the spectroscopic features observed on these extracted spectra (as expected for conventional raw MCARS spectra), they were processed by the MEM algorithm so as to extract the corresponding conventional Raman-like spectra (Figure 2C). We have highlighted in Figure 2C the vibrational modes that are most relevant for cell analysis in this spectral range, namely, CH_2_ symmetric (2,845 cm^−1^) and = C-H (3,005 cm^−1^) stretching, mainly associated to lipids (red vertical bars), CH_3_ symmetric (2,930 cm^−1^) and C-H aromatic (3,056 cm^−1^) stretching, mainly associated to proteins (green vertical bars), and O-H symmetric stretching (3,200 cm^−1^) of water (blue vertical bar). It is reminded that no vibrational signature of DAPI is expected in the high wavenumber (C-H stretching) region (Krause et al., 2007).

It appears from Figure 2C that components #1 and #4 are characterized by a high water content, concomitantly with the presence of proteins (for component #1) and lipids (for component #4). Components #2 and #5 are clearly dominated by the protein content but differ from each other by the lipid content (weak in component #2 and absent in component #5). Last, component #3 stands out by a strong lipid content, together with proteins. Therefore, from the spectroscopic viewpoint, the chemical species behind these components exhibit five different proportions of lipids, proteins, and water. This spectroscopic and chemical information of a given spectral contribution can then be related to the associated concentration map in order to observe the spatial distributions and biochemically investigate the intra- and extracellular contents.

In the following, we perform such biochemical study by simply using the spectral contributions extracted by the MC^2^ approach, that is, without phase retrieval computation by the MEM. To this aim, we have replicated and slightly shifted the vertical bars of Figure 2C onto Figure 2B, taking into account the spectral shift of peaks that occurs between raw CARS spectra and MEM-processed ones (Freudiger et al., 2008; Capitaine et al., 2018). In that respect and in a first approach, the spectroscopic analysis can be made from the MC^2^ spectra by appreciating the presence of CARS peaks in the different spectral channels indicated by the vertical bars. A more thorough interpretation is to consider the significance (mathematically, the slope and the amplitude) of the dispersive lines associated to these peaks. This is particularly useful when the CARS peak is not apparent, due to an insufficient ratio between vibrationally resonant and non-resonant contributions, as in the case of CH_3_ symmetric stretching for spectral contribution #3 (Figure 2B).

Finally, the fixed interphasic HEK293 cell observed in Figure 2 will be considered as the reference cell throughout the rest of this work. As a supplemental validation of our approach, we also applied MCR to the reference cell dataset after a prior step of MEM processing, taking into account the impact of the NRB spectrum source (here, the raw MCARS spectrum of the solvent) and of the baseline detrending (corresponding to the dark noise/background related to the spectrometer). Supplementary Figure S1 shows the evolution of the final outputs, according to these different processing steps. In this figure, the results obtained in the last case (NRB normalization combined with background subtraction) can thus be compared with MC^2^ results (first row). From the spectral point of view, we stress the obvious quality difference of Im{*χ* ^(3)^} spectra plotted in Figure 2C (MEM applied to the five spectra extracted by MCR) and Supplementary Figure S1 (MCR applied to all spectra first processed by MEM). Moreover, the Im{*χ* ^(3)^} spectra of Figure 2C demonstrate that MCR performs well with non-linear CARS intensity, since both the spectral shape and the exhibited vibrational information are consistent with Raman-like spectra and with the images of Figure 2A, respectively.

### 3.2 Comparative analysis of fixed cells in interphase (G_1_/S)

**FIGURE 3:**
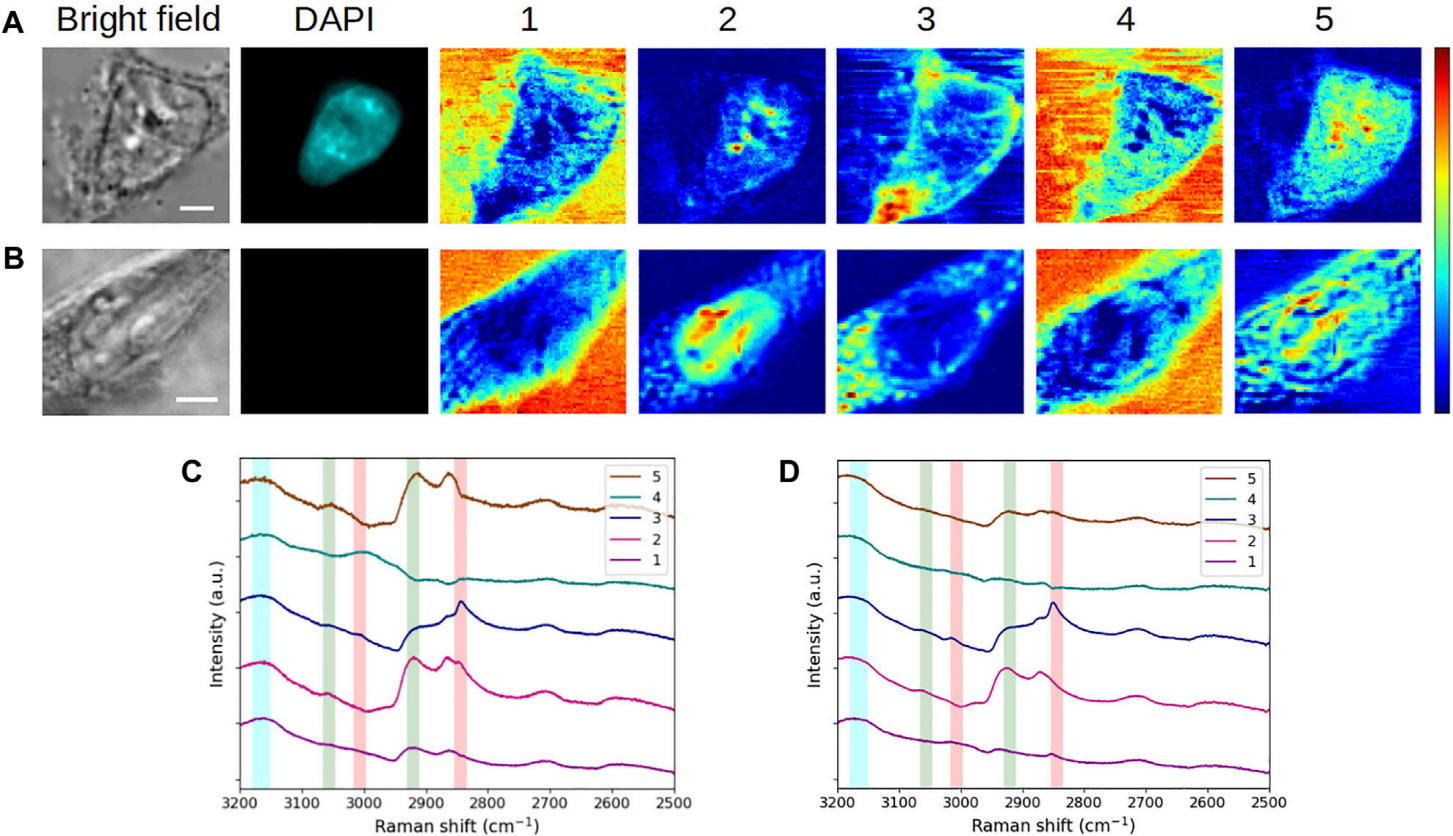
Comparative analysis of fixed **(A,C)** HEK293 and **(B,D)** C2C12 cells in interphase (G_1_/S). **(A,B)** Bright-field and fluorescence images and concentration maps obtained for the five computed components. The scale bar corresponds to 5 μm. **(C,D)** Spectra corresponding to **(A,B)**, respectively. Vertical bars highlight the presence of lipids (red), proteins (green), and water (blue). After fixation, the HEK293 cell was stained with DAPI.

In this part of the work, we assessed the robustness of the MC^2^ method by comparatively analyzing the HEK293 reference cell and a C2C12 interphasic unstained cell. Figure 3 shows the extractions obtained on these 2 cells. It should be noted that two separate MCR analyses were conducted on these two datasets.

When considering the concentration maps of the reference cell (Figure 3A), it is clear that components #1 and #4 (dominated by a high-water content, as already explained before) are mainly related to the aqueous extracellular environment. From a spatial point of view, component #2 is localized at the nucleus as depicted by its round shape, overlapping with DAPI staining. The high-concentration spots highlight specific areas that can reasonably be attributed to nucleoli in relation to DAPI staining together with bright-field images. Nucleoli are active nuclear subcompartments whose one of the main functions is to be the site for the initial steps of ribosome biogenesis. This implies the presence of proteins linked to the transcription machinery and post-transcriptional modifications by small nucleolar ribonucleoproteins in the dense fibrillary component. The rRNA assembly with the ribosomal proteins then occurs in the granular component of the nucleolus (Pederson, 2011). This is thus in agreement with the high protein content emerging in spectral profile #2 (Figure 3C). Regarding component #3, it is concentrated in an area corresponding to the plasma membrane (or its very close periphery) and it is found in the cytoplasm as well. This is in line with the chemical species highlighted by the associated spectrum, that is, a lipid-rich content with the presence of proteins. Finally, component #5, which corresponds to a high protein content and an absence of lipids, is located throughout cell nucleus and cytoplasm, with a higher concentration in the nucleus and near nucleoli.

Overall, the extracted spectra of HEK293 and C2C12 cells (Figures 3C,D, respectively) exhibit a reasonable stability when they are observed pairwise for each component (except for component #5, which will be examined later). Accordingly, the concentration maps (Figures 3A,B) reveal a comparable spatial distribution of the signal between both cells for the first four components. Hence, with a high-water content, components #1 and #4 highlight the extracellular milieu. The observations made for component #2 in the case of the reference cell are applicable to the second cell, that is, a high protein content (with more or less lipids) emphasizes the nucleus and nucleoli (visible in bright-field for both cells). Regarding component #3 (lipid-rich content with the presence of proteins), the distribution of the signal in the cytoplasm of the C2C12 cell is more or less punctiform, suggesting that the related cytoplasmic structures correspond to lipid droplets. Lipid droplets are organelles consisting in a phospholipid monolayer surrounding a core composed of neutral lipids. They are well described in C2C12 premyoblastic cells (Billecke et al., 2015; Ichimura et al., 2015), and their abundance in myoblast cytoplasm was recently associated with a facilitated (induced) differentiation into myotubes (Tan et al., 2021). Moreover, in both cells, a portion of component #3 appears to surround the nuclear structure and could be related to the nuclear envelope. For all these reasons, component #3 seems to relate to cellular membrane constituents such as phospholipids.

In the case of component #5, a variability is observed between both cells. For the reference HEK293 cell, this component represents a pure protein content in the cytoplasm and, more abundantly, in the nucleus, which is difficult to assign to a particular cellular compartment. For the C2C12 cell, spectrum #5 (Figure 3D) reveals a mixed content of proteins, lipids, and water. In the corresponding concentration map (Figure 3B), the signal is less diffuse and more reticular than for the HEK293 cell. This would suggest that endoplasmic reticulum (ER) is highlighted, assuming that lateral resolution is not sufficient to resolve individual ER layers and that the seeming presence of signal in the nucleus is due to positioning the focal plane at the nucleus periphery. However, further experiments would be necessary to support this assertion, including the specific labeling and fluorescence imaging of the ER. Anyhow, in both cases, the concentration map of component #5 is complementary to the other four maps. Regarding the variability of the spatial distribution between HEK293 and C2C12 cells, it can be hypothesized that component #5 is specific to the cell line, given that the cells studied here belong to distinct types with very different physiological functions.

### 3.3 Comparative analysis of fixed cells in interphase (G_1_/S) and mitosis (prophase)

**FIGURE 4:**
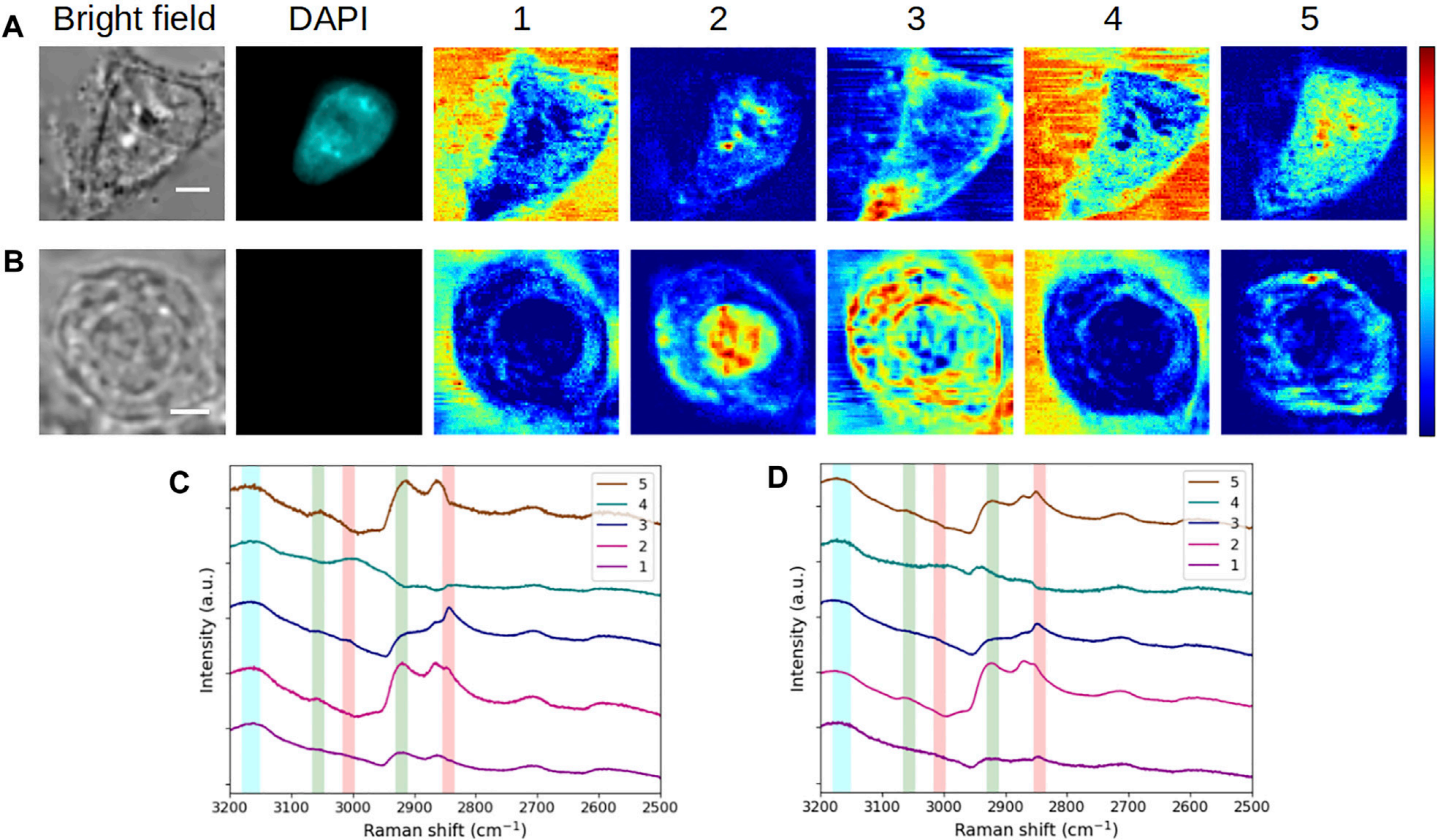
Comparative analysis of fixed HEK293 cells in **(A,C)** interphase (G_1_/S) and **(B,D)** mitosis (prophase). **(A,B)** Bright-field and fluorescence images and concentration maps obtained for the five computed components. The scale bar corresponds to 5 μm. **(C,D)** Spectra corresponding to **(A,B)**, respectively. Vertical bars highlight the presence of lipids (red), proteins (green), and water (blue). After fixation, the interphase cell was stained with DAPI.

Next, we compared the reference interphasic cell with an HEK293 unstained cell that was fixed in mitosis (prophase), as displayed in Figure 4. As previously, MCR analyses were performed separately.

Again, it is interesting to see that a certain stability is maintained at the spectral level between both cells for components #1–#4. Accordingly, in the concentration maps of the mitotic cell (Figure 4B), the extracellular environment is disclosed by components #1 and #4. Component #2 (high protein content) shows a strong and rather homogeneous signal at the place of nucleus, in agreement with the nuclear rearrangements occurring during prophase. The observed intensity variations can be correlated with the level of chromatin condensation. The concentration map of component #3 (lipid-rich content with proteins) highlights concentric structures looking like membranes, the outermost layer corresponding to the plasma membrane. During mitosis, a major rearrangement of membrane structures takes place and is essential for the completion of a proper cell division leading to fully functional daughter cells (recently reviewed in Carlton et al., 2020). The fractionation of some of the organelles such as the ER and the Golgi apparatus, disassembled during late prophase (or prometaphase), leads to a new membrane organization with a modification of the protein/lipid ratio in the resulting membranes. Notably, an important part of the membrane components is bound to the plasma membrane. It is interesting to note that the concentric lamellar signal observed in concentration map #3 suggests such an organization, which would be consistent with the division state of the studied cell, visually estimated in prophase to late prophase.

Regarding component #5, we notice a divergence in the spectra and concentration maps corresponding to the interphasic and mitotic cells, respectively. We can assign this divergence to the different physiological states of the cells. In the case of the mitotic cell, the extracted spectrum displays a mixed protein-lipid content and the resulting concentration map exhibits a specific, polarized, and intracellular structure in the cytoplasm. As seen in Supplementary Figure S2A, this structure (plotted in green here) is entangled with component #3 (plotted in red). The same trend is visualized for another (late) prophase cell (Supplementary Figure S2B), with an even more distinct polarization of component #5. These observations would suggest that this component might be linked to the mitotic spindle of the cells. The results obtained for an early-metaphase cell (Supplementary Figure S2C) consolidate this hypothesis, as well.

### 3.4 Comparative analysis of interphase (G_1_/S) fixed and living cells

**FIGURE 5:**
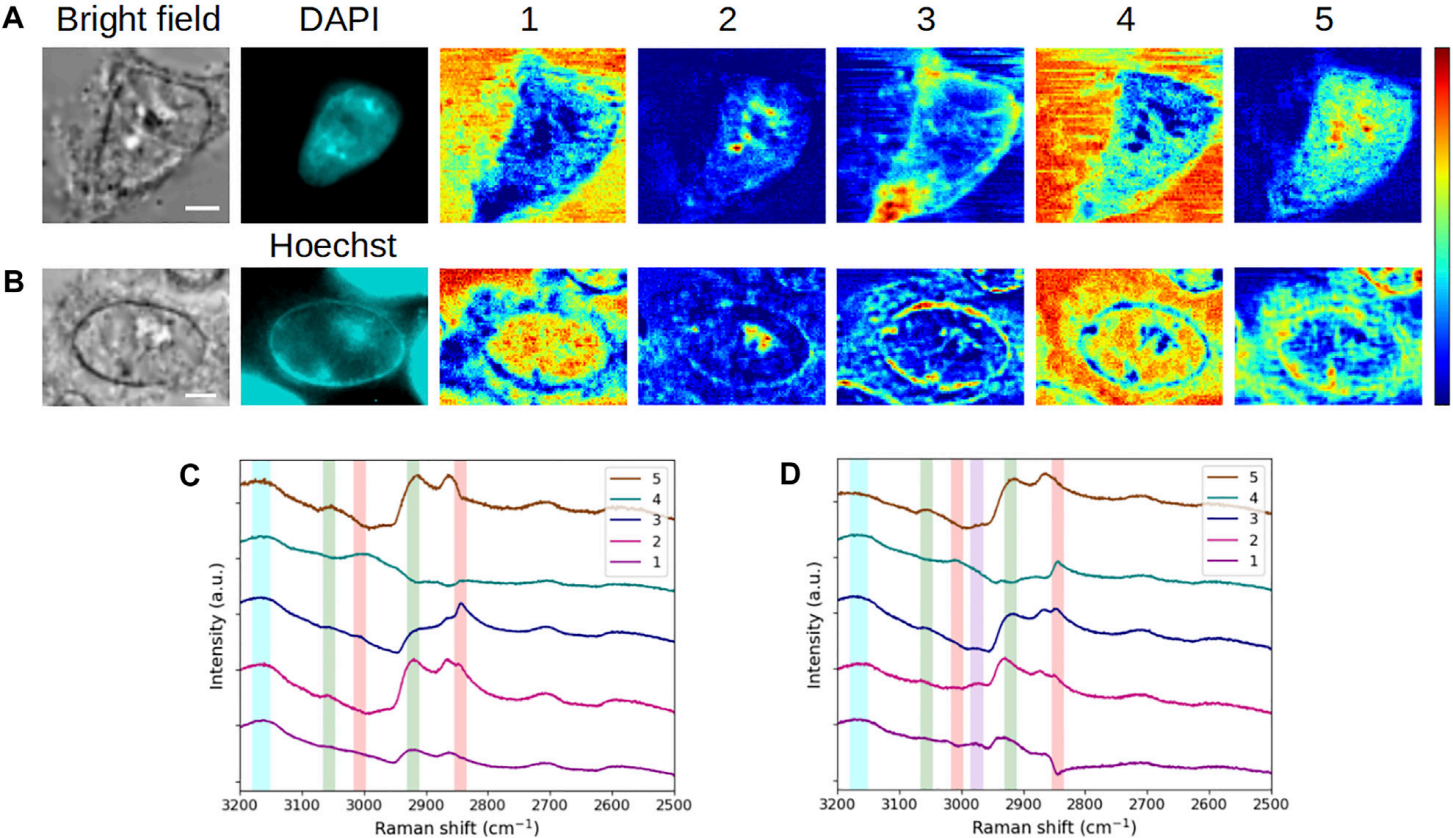
Comparative analysis of interphase **(A,C)** fixed and **(B,D)** living HEK293 cells. **(A, B)** Bright-field and fluorescence images and concentration maps obtained for the five computed components. The scale bar corresponds to 5 μm. **(C, D)** Spectra corresponding to **(A, B)**, respectively. Vertical bars highlight the presence of lipids (red), proteins (green), DNA/RNA (violet), and water (blue). The fixed and living cells were stained with DAPI and Hoechst 33342, respectively.

Cell fixation prior to *in situ* labeling and/or the use of several imaging methods have non-negligible effects on cell structure. These effects differ according to the fixation reagent. For example, formaldehyde, routinely used, can react with numerous functional groups of macromolecules, especially inducing protein or protein-DNA cross-linking (Hoffman et al., 2015). The consequences of fixation from a chemical standpoint are accompanied by the alteration of cell mechanical properties, which were investigated in Kim et al. (2017) by means of atomic force microscopy and scanning ion conductance microscopy. For their part, Hobro and Smith (2017) could use Raman spectroscopic imaging and PCA to evaluate several fixation methods. In this context, we sought to assess our approach for the study of cell alterations induced by fixation. In that way, Figure 5 presents the concentration maps and the associated spectra, resulting from the application of the MC^2^ method to the reference cell (Figures 5A,C), fixed with PFA, and a living HEK293 cell (Figures 5B,D).

First, some common features persist when comparing the concentration maps of the 2 cells. Components #1 and #4, dominated by the water content, again points out here the extracellular environment. Nucleoli visualization is still found in component #2, while component #3 discloses membrane structures. However, we can notice some obvious differences between both cells, which are described later.

In the living cell, component #1 highlights not only the extracellular milieu but also the nucleus, which is visible for neighbor cells in the field of view, as well. In addition to the nucleus, the cytoplasm is shown by component #4, which is spectrally characterized by the combined presence of water and lipids. Interestingly, this component spotlights the border of the nucleus and the nucleoli as low-signal areas, indicating that water and lipids are not found together in these areas (the sole lipids being expected at the nuclear envelope and non-expected in the nucleoli). Overall, the presence of the intracellular aqueous content is not observed for the fixed cell. This would be consistent with a loss of cellular content due to the fixation that may induce shrinkage, especially when performed at room temperature.

In the case of component #2, besides nucleoli, the border of the nucleus (the nuclear envelope) is highlighted in the living cell, in agreement with bright-field and Hoechst 33342 fluorescence images. A further investigation of spectrum #2 brings out some vibrational information in the channel plotted in violet (Figure 5D). This contribution is assigned to CH_3_ antisymmetric stretching in the 2,960–2,980 cm^−1^ range (Matthews et al., 2010). In Supplementary Figure S3, we have compared the spectra of component #2 obtained for all fixed and living cells considered so far, including both raw and MEM-processed spectra. This figure displays a stronger contribution of the interphase living HEK293 cell in the highlighted 2,960–2,980 cm^−1^ channel. Following Lu et al., 2015, this contribution can be attributed to DNA in live cells and can thus be associated with a structure linked to the nuclear envelope in the present case (Figure 5B, map #2). Indeed, during interphase, heterochromatin binds indirectly to the nuclear envelope by means of proteins involved in tethering chromatin. The absence of such a perinuclear signal in the fixed cell (Figure 5A, map #2) would be consistent with the chemical mechanism of action related to cell fixation with PFA.

Regarding component #3, in the living cell, the cytoplasm and the nuclear border clearly appear. Taking into account the combined presence of lipids/proteins and the reticular aspect of the signal in the cytoplasm, one can suggest that the revealed structures correspond to the nuclear envelope in contiguity with the rough ER. This assumption is supported by the weak contribution of CH_3_ antisymmetric stretching to component #3 (Figure 5D, spectrum #3, violet channel), which is assigned to ribosomal RNA in the cytoplasm (Lu et al., 2015). Finally, component #5, dominated by proteins and exempt from lipids, discloses the inner border of the nuclear envelope, likely corresponding to the nuclear lamina and the cytoplasm, likely due to the presence of resident soluble proteins in the latter. The absence of lipids in this component results in a “double ring” signal, localized on both sides of the nuclear envelope.

**FIGURE 6:**
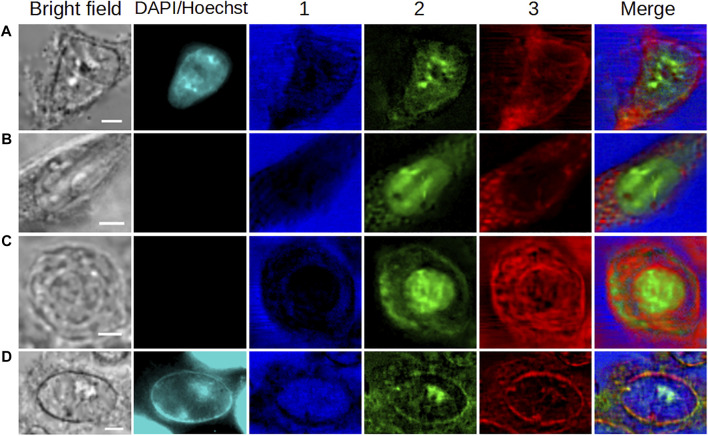
Overview of the application of the MC^2^ approach to single-cell imaging in different cases. **(A,B)** Fixed interphase cells. **(C)** Fixed mitotic cell. **(D)** Living interphase cell. Images in 1,2, and 3 are the concentration maps of components #1, #2, and #3 extracted from Figures 2A, 3, 4, 5B, respectively, and plotted in false blue/green/red colors. The scale bar corresponds to 5 μm.

In order to illustrate the potential of the MC^2^ approach for single-cell imaging, Figure 6 summarizes the results obtained with HEK293 and C2C12 cells in the different physiological states (fixed interphase, fixed mitotic, and living interphase cells). In this figure, we plotted the concentration maps of components #1, #2, and #3 from Figures 2A, 3, 4, 5B in false colors, specifically in blue, green, and red, respectively. The merge column shows the overlay of these three maps, illustrating the complementarity of the extracted components under each biological condition. Moreover, an easier comparative analysis of all results can be made from Figure 6.

For a further validation of our approach, the workflow of Supplementary Figure S1 (NRB normalization + background subtraction + MEM + MCR) was applied to the four cells of Figure 6. Output maps and spectra are available in Supplementary Figures S4 and S5. Generally speaking, combining MEM and MCR leads to more noisy images and spectra, due to additional processing steps and thus potential loss of information. In Supplementary Figure S4, the parallel representation of “MCR” and “MEM + MCR” results allows to validate our new approach, especially by comparing the high-protein and high-lipid content channels (second and third columns, in each case). However, even if our approach is workable and concentration maps are quite similar in this figure, we draw attention to the fact that, rigorously, the method is not expected to be quantitative since the raw CARS signal is not proportional to the concentration.

### 3.5 Analysis of living cells overexpressing TrkB and treated or not with brain-derived neurotrophic factor

**FIGURE 7:**
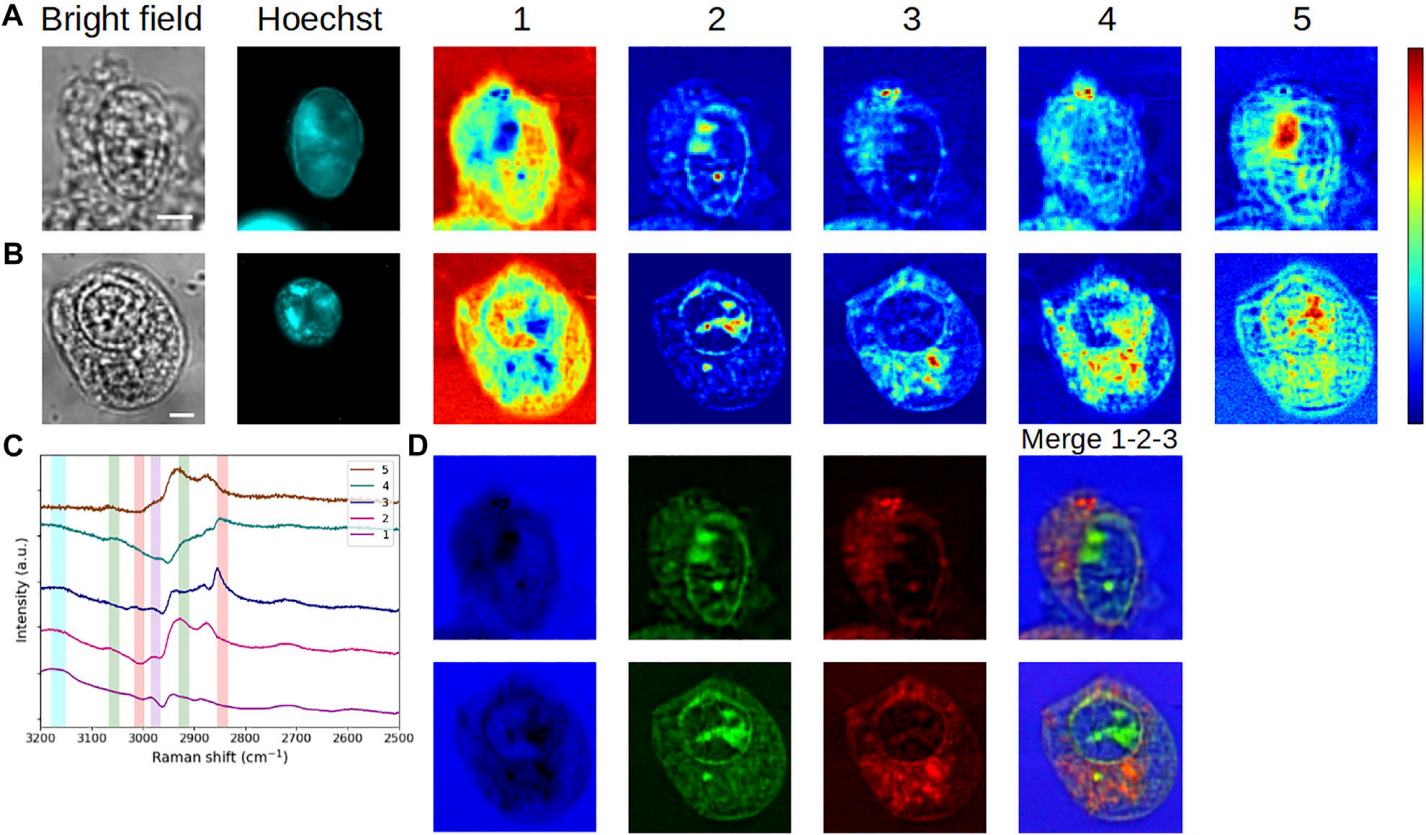
Analysis of living cells overexpressing TrkB cancer-related protein, in the presence or absence of its ligand BDNF. Application of the MC^2^ approach to the analysis of a cell that was incubated without BDNF, with its **(A)** concentration maps and **(C)** corresponding spectra, considered as reference spectra. Vertical bars highlight the presence of lipids (red), proteins (green), DNA/RNA (violet), and water (blue). **(B)** Concentration maps of a cell that was incubated with BDNF, obtained by the projection of its dataset onto the reference spectra. **(D)** False-color images corresponding to concentration maps #1 (blue), #2 (green), and #3 (red). Both cells were incubated without/with BDNF during 72 h and stained with Hoechst 33342. The scale bar corresponds to 5 μm.

Last, we evaluated the robustness and the applicability of the MC^2^ method for detecting changes in cells where a variable was introduced. For this purpose, we analyzed the datasets of two living HEK293 cells overexpressing the TrkB receptor at a basal level or activated by the introduction of its ligand, BDNF, in the culture medium for 72 h (Figure 7). In this case, we used the spectra calculated for the non-treated cell (Figure 7C) as a reference, and then we projected both datasets onto this base to construct the concentration maps of the non-treated (Figure 7A) and TrkB-activated (Figure 7B) cells. In addition, both cells can be visualized in false colors for components #1–3, as displayed in Figure 7D.

As an initial investigation, the information contained in components #1–3 is rather similar to that of the previously studied living HEK293 cell. Specifically, in both cells, some water appears in the nucleus (except the nucleoli), the nucleoli, and the nuclear border are highlighted, as well as the lipid content in the cytoplasm. As previously, the DNA contribution is found in spectrum #2, as confirmed in Supplementary Figure S2 (green spectra). Concerning component #3, given the punctiform distribution of the corresponding intracytoplasmic signal and considering the shape of spectrum #3, it is stressed that this component exhibits lipid droplets in both cells. Thereupon, the apparent larger accumulation of lipid droplets in the TrkB-activated cell would be related to a modification of the lipid metabolism, as suggested by Guerenne-Del Ben et al., 2020. Further than this work, here the comparative study of the non-treated and treated cells is substantially facilitated by the great complementarity between components #1–3. In the case of component #4, the obvious increase of high-intensity spots in the second cell may also correlate with the alteration of the lipid metabolism.

Finally, for component #5, the signal is distributed throughout the nucleus and the cytoplasm of both cells, but a more reticular structure is observed in the non-treated one. In both cases, an intense spot is visible in the nucleus, partially overlapping with nucleoli and low-water content areas. Another feature is the presence of the signal, in the TrkB-activated cell, at the place of the plasma membrane. Yet, proteins constitute the main vibrational signature in spectrum #5, which furthermore differs from other spectra by the complete lack of contribution in the water region. Then, this high protein content near the plasma membrane may reflect the consequences of cell exposure to the growth factor, and particularly the activation of TrkB by BDNF at the cell surface. At this step, it is more difficult to interpret the intracellular content revealed by component #5.

### 3.6 Comparison between MC^2^ and MC^2,S^ methods applied to the reference cell

We have seen in the previous sections that the MC^2^ approach allows to extract interesting biological information from the MCARS spectra. Nevertheless, we should not forget that only the spectral data were exploited to generate these maps and that the spatial information was not exploited in this approach. We thus developed the MC^2,S^ method in order to take into account simultaneously the spectral and spatial dimensions, to refine the extraction of components and to perform cell segmentation.

As mentioned in Section 2, the length penalty *µ* is an important parameter to set (between 0 and 1) in the MC^2,S^ approach. Several values of *µ* were tested for the reference cell, as illustrated in Supplementary Figure S6. We could then study the evolution of the cell contour according to *µ* variation, for each of the five components extracted by MCR^S^. Small values of *µ* lead to cropping the bottom-left part of the cell, particularly in the case of components #1 and #5 (arrow heads, Supplementary Figure S6). From *µ* = 0.35, this bottom-left curve becomes comparable to what is observed without using the segmentation constraint. Higher values of *µ* induce an oversegmentation, visible for components #1, #3, and #4 on the upper side of the cell (full arrows, Supplementary Figure S6). For these reasons, we set the value of *µ* to 0.35.

**FIGURE 8:**
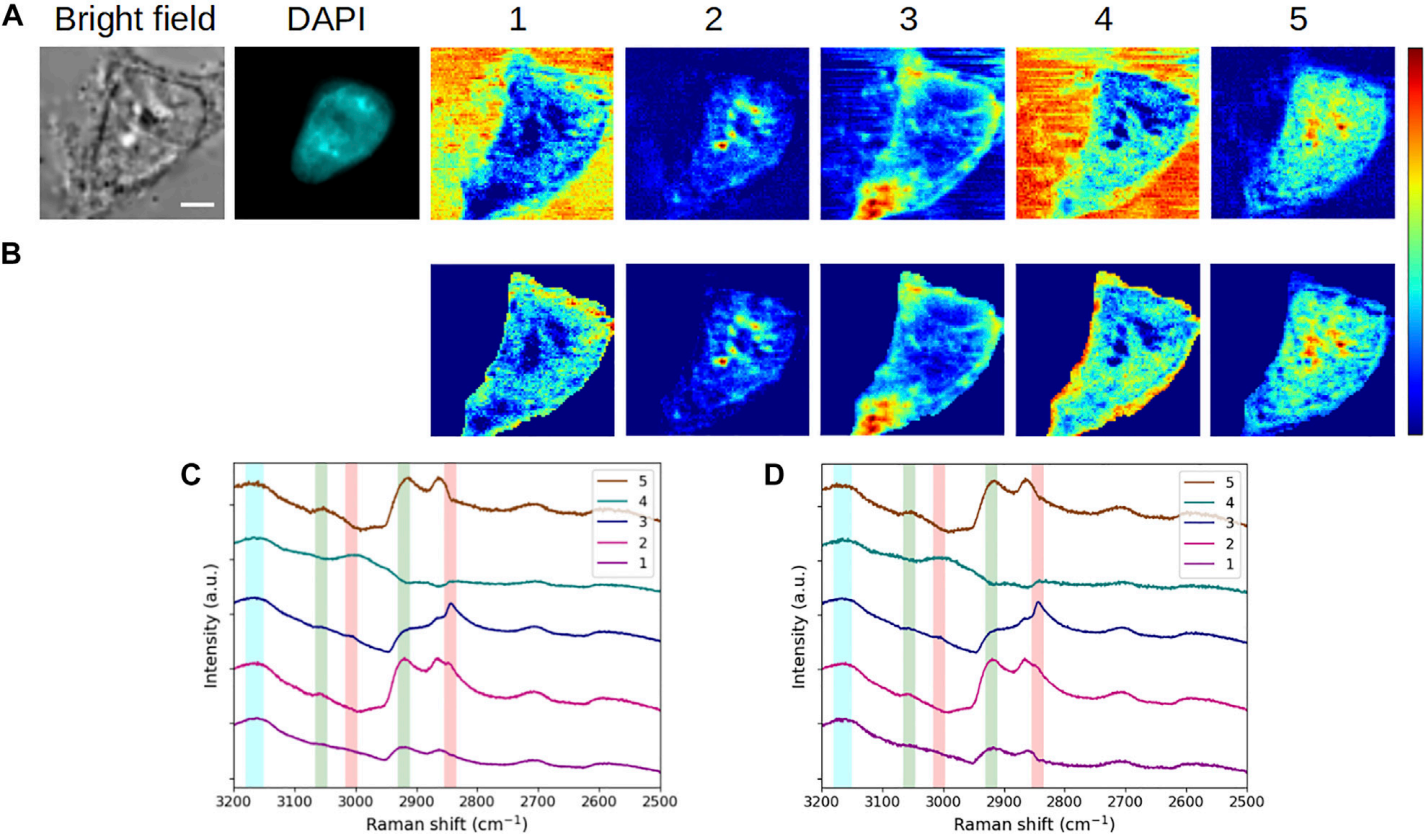
Analysis of a fixed HEK293 cell in interphase (G_1_/S) without or with segmentation. Concentration maps and corresponding spectra obtained by means of **(A, C)** MC^2^ and **(B, D)** MC^2,S^ approaches. The scale bar corresponds to 5 μm. Vertical bars in **(C, D)** highlight the presence of lipids (red), proteins (green), and water (blue). After fixation, the cell was stained with DAPI.

Then, to appreciate the impact of the addition of the segmentation constraint to the MCR framework, both MC^2^ and MC^2,S^ methods were applied to the reference cell. The results are shown in Figures 8A,C and in Figures 8B,D, respectively.

The vibrational modes of each extracted spectrum were not modified by the introduction of the segmentation constraint, though some noise appeared on the spectral profiles. Regarding concentration maps, the value added by the new constraint is undeniable since the cell is clearly delimited from the background under these new conditions. In addition, the improved contrast within the cell allows a better rendering of intracellular structures. The most obvious changes are observed in concentration maps #1 and #4. In these maps, the extracellular milieu was initially the main contributing element. By using the segmentation constraint in the MCR process, the resolution is tightened on the intracellular area, resulting in a more accurate extraction of the aqueous content inside the cell. As a consequence, the main contributions in concentration maps #1 and #4 now appear in the inner periphery of the plasma membrane (this is particularly true for component #4) and, secondarily, in the nucleus and cytoplasm. Beyond the investigation of intracellular water, MC^2,S^ would be of interest for the study of elements that are prominent in the cell environment.

### 3.7 Application of MC^2^ and MC^2,S^ methods to the analysis of cell clusters

**FIGURE 9:**
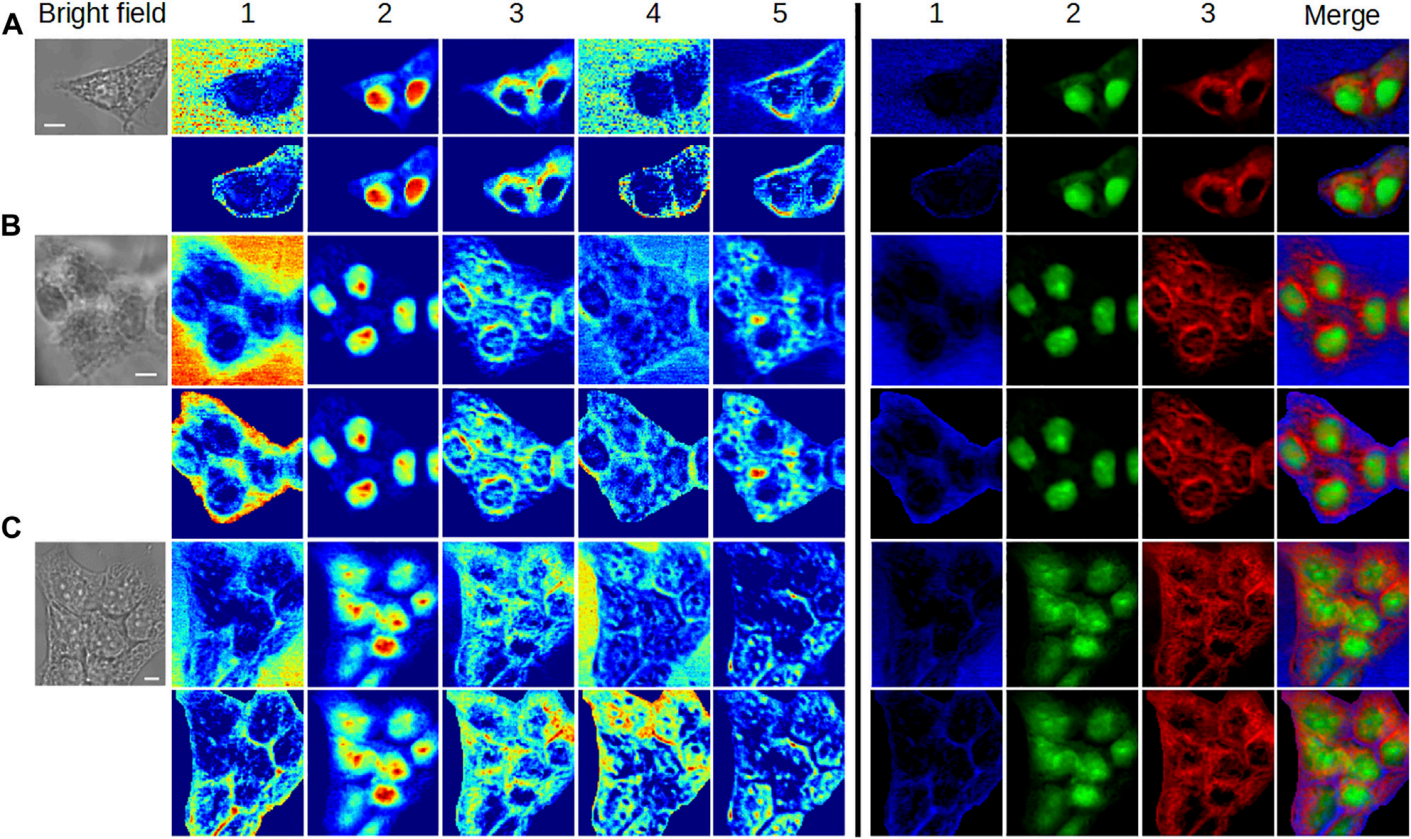
Application of MC^2^ and MC^2,S^ approaches to the analysis of cell clusters. **(A)** 2-cell cluster, **(B)** 5-cell cluster, and **(C)** 8-cell cluster. For each cluster, the concentration maps computed by MC^2^ and MC^2,S^ are plotted in first and second rows, respectively, using two different colormaps. The scale bar corresponds to 5 μm. The corresponding spectra are available in Supplementary Material.

Three different unlabeled, fixed, HEK293 cell clusters were analyzed to assess the robustness of MC^2^ and MC^2,S^ methods and their applicability to more complex situations (Figure 9). The corresponding spectra are given in Supplementary Figures S7–S9.

When looking at the concentration maps obtained by the MC^2^ approach, we can notice a good reproducibility in the extraction of components for each cell cluster, with respect to previous results.Specifically, components #1 and #4 reveal the extracellular environment. Component #2 is associated with nuclei/nucleoli. Component #3 is mostly cytoplasmic and highlights a perinuclear structure (obvious in 2-cell and 5-cell clusters), probably made up of intracellular membranes belonging to the ER. Component #5 allows to visualize other cytoplasmic elements distributed asymmetrically within the cells.

The application of the MC^2,S^ method provides the suitable segmentation of each cell cluster and a better visualization of intracluster structures. This benefit is noticeable in the false color representation of concentration maps #1, #2, and #3. In particular, it is interesting to see the presence of water in the intercellular space (5-cell and 8-cell clusters) and on the periphery of the clusters. Of course, the study of aqueous content would be supplemented by taking component #4 into account and by investigating the contribution of vibrational modes reflecting the presence of other chemical species. The MC^2,S^ approach would thus be of interest to determine the state of water, namely, free water or water hydrating macromolecules.

### 3.8 Statistical analysis of cell segmentation

The accuracy of the segmentation included in the MC^2,S^ analysis was evaluated by calculating the Dice similarity coefficients that are well recognized as a valuable metrics (Hermsen et al., 2019; Eelbode et al., 2020). Dice coefficients estimate the spatial overlap between the ground truth, corresponding in our case to a manual segmentation made by a cell biologist, and the segmentation mask generated by the MC^2,S^ method. They are defined by DC (Ground truth, MC^2,S^ segmentation) = 2 (|Ground truth|∩|MC^2,S^ segmentation|)/(|Ground truth|+|MC^2,S^ segmentation|), where ∩ is the intersection. Dice coefficients are comprised between 0 and 1, 0 being a total absence of overlapping, and 1 a total overlap between manual and automatic segmentations. We calculated the Dice coefficients for a total of 18 comparisons that correspond to cells or clusters included in Guerenne-Del Ben et al., 2019 and to unpublished results obtained in the context of the cited work. These comparisons were classified according to four categories, as shown in Supplementary Figure S10 (interphase fixed cells), Supplementary Figure S11 (mitosis fixed cells), Supplementary Figure S12 (interphase living cells), and Supplementary Figure S13 (fixed cell clusters).

**FIGURE 10:**
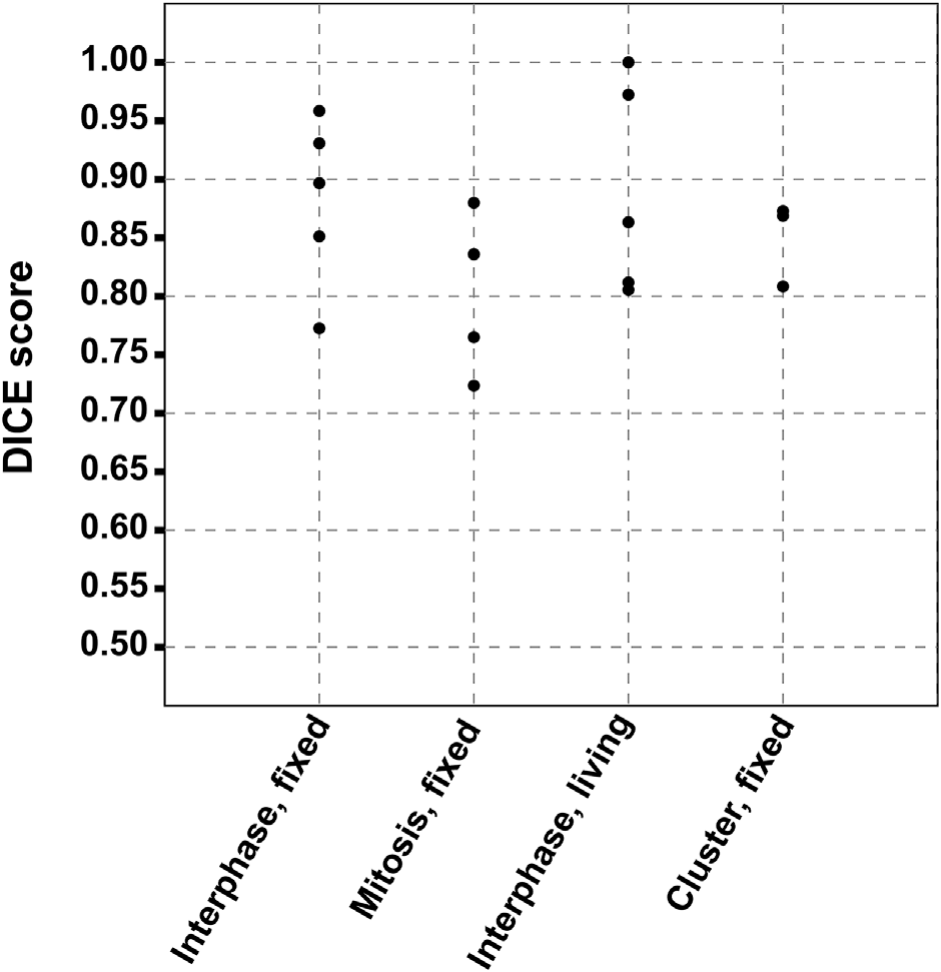
Dice coefficients according to the physiological state of the cells. The similarity was calculated between the ground truth, corresponding to the manual segmentation from bright-field images and the segmentation mask generated by the MC^2,S^ method.

Overall mean of Dice coefficients was 0.86 ± 0.07. In light of this result, MC^2,S^ segmentation can be considered of good quality, all the more so as manual segmentation from bright-field images is a source of error and CARS measurement differs from bright-field by its optical sectioning capability (causing lateral shift and/or size modification between both modalities, as seen in several cases). Finally, we plotted in Figure 10 the calculated Dice coefficients, according to the physiological state of the cells. Following the *p*-value analysis of these data (*p* > 0.05), no significant difference was observed between the different cell categories, highlighting the robustness of the proposed method toward the cell morphology.

## 4 Discussion

### 4.1 Determination of the number of components used in multivariate curve resolution

Here, we discuss the strategy that we adopted to set the number of components to K=5 .

From the signal analysis point of view, we consider that the analyzed MCARS hypercube is created by various sources of information, each of them having an internal structure that can be exploited. There are many methods in the literature for solving this source separation problem. A first family of methods relies on stochastic assumptions, the main approach in this field being PCA. The latter consists in diagonalizing the covariance matrix to build a projection basis and thus decorrelate the data (specifically, the second-order moments). A second family of methods aims at providing the sparsest approximation, allowing to reconstruct the initial dataset as best as possible. A typical example is the matching pursuit (Shi et al., 2013). We position our approach in this second family of methods. As previously mentioned, in the case of MCR, the C matrix can be interpreted as the projection of original data onto a new vector basis defined by $S^{T}$ , which contains K rows (i.e., K components). Whichever method is chosen, a difficult question is to estimate the size of the projection space ensuring the selection of the informative components from the analyzed signal.

For the PCA approach, most of the estimators work exclusively from the eigenvalues of the covariance matrix. It is usual to use a screen test, which consists in detecting the existence of a significant decay in the eigenvalues diagram for determining the rank (the number of eigenvectors to retain). In practice, this method is not always reliable because of its subjectivity. Another method is to use the approximation error to determine the best choice of the number of components: during the iterative construction of the projection basis, the Euclidean norm of the approximation error is used in the stopping criterion. In the case of a linear transformation like conventional PCA, these two methods of rank selection are actually equivalent.

For the MCR approach, it is common practice to determine the dimension of the projection space by studying the eigenvalues of the covariance matrix—by means of PCA—before the MCR computation. However, in our case, we focus on building the best approximation of the initial dataset. Therefore, we introduce the use of the LOF to evaluate the quality of the MCR decomposition according to the value of K . In this context, the LOF mean and standard deviation, $\mu_{LOF}$ and $\sigma_{LOF}$ , respectively, were calculated for the whole group of datasets considered in the present work. The elbow method (Thorndike, 1953) was then applied to determine the best value of K . Table 1 shows $\mu_{LOF}$ and $\sigma_{LOF}$ with K varying from 1 to 15. The corresponding curve is plotted in Supplementary Figure S14. According to the elbow method, we derive 4≤K≤6 with $0.243\geq \mu_{LOF}\geq 0.202$ and $0.443\geq \sigma_{LOF}\geq 0.361$ . Given that K=5 provides new information compared to K=4 , whereas K=6 only brings redundancy, and the number of components is set to K=5 . As a comparison, by applying the criterion of decay of the eigenvalues, we obtain K=2 , which is clearly insufficient in view of the information effectively contained in the datasets. Thus, we suggest that our strategy is appropriate to realize the unsupervised exploration of the different cell samples.

**TABLE 1:** LOF evolution according to the number of components. First row is the number of components K extracted by MCR, second row is the LOF mean $\mu_{LOF}$ (in percent), and third row is the LOF standard deviation $\sigma_{LOF}$ (in percent). The corresponding curve is given in Supplementary Figure S7.

*K*	1	2	3	4	**5**	6	7	8	9	10	11	12	13	14	15
$\mu_{LOF}\left(\%\right)$	0.481	0.369	0.262	0.243	**0.214**	0.202	0.20	0.192	0.193	0.189	0.186	0.186	0.186	0.175	0.175
$\sigma_{LOF}\left(\%\right)$	0.771	0.592	0.463	0.443	**0.373**	0.361	0.365	0.356	0.363	0.362	0.355	0.354	0.355	0.327	0.327

The “elbow” points are underlined, and the selected value is in bold.

### 4.2 Robustness and relevance of the MC^2^ method for label-free cell imaging

For all the cells considered in this study, except those overexpressing TrkB, the first four MCR components are remarkably stable, highlighting cell common constituents (in both spectra and maps) and allowing a consistent comparison between the samples. Component #5 appears to be more specific to each studied individual condition and contains information related to this specificity, namely, cell type, step of the cell cycle, and (patho) physiological or metabolic status. In this sense, component #5 is highly relevant and confirms the capability of the MC^2^ method to extract peculiar information. However, as this component is specific, a direct comparison of different cells (regarding component #5) is not sufficient for a reliable analysis. Hence, the use of MC^2^ on a reference cell/condition and the subsequent projection of data obtained with different cells/conditions onto the reference spectra is particularly useful. This kind of data relativization is commonly used in biology, for example in metabolic activity assays, or for representing qRT-PCR data (ΔΔCT method). The choice of the reference cell/condition is then crucial, and modifying this choice allows to carry out a systematic and deeper analysis, considering the specificity of component #5. Of course, this approach is applicable to all components extracted by MCR. Furthermore, it is stressed that it is valid when only a unique variable is introduced in the experimental context (e.g. pharmacological treatment, ligand binding, *etc*.), as in the case of living cells overexpressing TrkB and treated or not with BDNF.

In view of the overall results presented in this work, the MC^2^ method shows its great potential in label-free cell imaging. First, the observations made here are consistent with the previous ones using largely the same datasets (Guerenne-Del Ben et al., 2019; Guerenne-Del Ben et al., 2020). Beyond that, we could make more accurate analyses and comparisons of experimental situations, thanks to the superior unmixing of cell constituents and the extraction of additional contributions. For instance, when comparing cells in different stages of the cell cycle, it was possible to propose a better estimation of the mitotic subphase (from late prophase/prometaphase to metaphase). New information came also from the comparison of the living cell with the fixed one regarding the loss of cellular content due to the fixation. Yet, the reader should note that these findings are based on the study of individual cells to establish a proof-of-concept, and that they cannot, at this step, be generalized to cell populations.

### 4.3 Refinement of intracellular/intracluster analysis by the addition of a segmentation constraint (MC^2,S^ method)

As displayed in Figure 8 for the reference cell, the MC^2,S^ method performs a good extraction of the cell from its environment and improves the image contrast within the cell, allowing to refine the intracellular analysis. The use of the segmentation constraint makes the LOF increase from 0.165 to 55.6% in this case, expressing that a major part of data (the extracellular milieu) was removed. Therefore, MC^2,S^ can be of particular interest for studying intracellular events linked to a component that is in abundance in the extracellular milieu. The aqueous content, associated with components #1 and #4, is a perfect illustration of this perspective, as seen in the corresponding concentration maps (Figure 8B). In that respect, it is worth noting that the study of “biological water” can raise different view angles and interests, depending on the involved disciplinary field (interestingly outlined in Jungwirth, 2015). Anyhow, we stress that the detailed knowledge of the water state and distribution in cellular compartments would have a high (patho) physiological relevance. Moreover, several optical approaches were recently introduced for intra/extracellular water imaging, based on MCARS (Nuriya et al., 2019), stimulated Raman excited fluorescence (Shi et al., 2019), or fluorescence lifetime measurement (Rao et al., 2019). In this context, we suggest that the MC^2,S^ approach could significantly contribute to the field by taking into account both spatial and spectral dimensions from data acquisition to numerical processing. Obviously, our approach can be extended to the O-H stretching and fingerprint regions, as we already demonstrated ultra-multiplex CARS with 500–4,000 cm^−1^ coverage in previous works (Kano et al., 2019; Nuriya et al., 2019; Kaneta et al., 2021).

Finally, the potentialities offered by the MC^2,S^ method are confirmed in the case of cell clusters, whatever number of cells is considered. Here, the extraction of information is refined inside the cluster and, in particular, the water content can be clearly visualized between the cells forming the cluster. These results show that the MC^2,S^ method is also applicable to the study of biological tissues.

To conclude, this work establishes MC^2,S^ as a new methodology for MCARS hyperspectral cell imaging and segmentation, based on a simple, unsupervised workflow without any spectrum-to-spectrum phase retrieval computation. Such easy-to-use methodology, combined with the constant simplification of MCARS instrumentation, should substantially participate in disseminating coherent Raman technologies into the biomedical field.

## Data Availability

Publicly available datasets were analyzed in this study. This data can be found here: https://gitlab.xlim.fr/datacart/mc2s.
